# Time in target range for systolic blood pressure and stroke in people with and without diabetes: the Kailuan prospective cohort study

**DOI:** 10.3389/fendo.2025.1537343

**Published:** 2025-05-14

**Authors:** Yuxin Yang, Shouling Wu, Jingdi Zhang, Yang Liu, Mingjie Yin, Zhenyu Huo, Shuohua Chen, Guodong Wang, Yao Xiao, Yue Wang, Yuanyuan Li, Jiawen Deng, Tingting Geng, Hong Zhang

**Affiliations:** ^1^ Department of Neurology, Shengjing Hospital of China Medical University, Shenyang, China; ^2^ Department of Cardiology, Kailuan General Hospital, Tangshan, China; ^3^ Department of Epidemiology and Biostatistics, School of Public Health, North China University of Science and Technology, Tangshan, China; ^4^ Department of Epidemiology and Biostatistics, School of Public Health, Peking University, Beijing, China; ^5^ Department of Internal Medicine, Hebei Medical University, Shijiazhuang, Hebei, China; ^6^ Department of Epidemiology and Biostatistics, Ministry of Education Key Laboratory of Environment and Health, School of Public Health, Tongji Medical College, Huazhong University of Science and Technology, Wuhan, Hubei, China

**Keywords:** diabetes, Kailuan study, stroke, systolic blood pressure, time in target range

## Abstract

**Objective:**

Systolic blood pressure time in target range (SBP-TTR) is an independent risk factor for stroke. We aimed to investigate the associations of SBP-TTR with stroke among participants with or without diabetes using data from the Kailuan study.

**Methods:**

We included 28,591 participants [mean age, 57.5 years; 83.8% men; 23.2% with diabetes] from the Kailuan Study. Cox proportional hazards regression models were used to estimate the hazard ratios (HRs) and 95% confidence intervals (95% CIs) of SBP-TTR on stroke in individuals with and without diabetes.

**Results:**

After a median of 8.7 years follow-up, 2,206 stroke cases occurred. Among participants with diabetes, those with SBP-TTR 75%–100% (HR [95%CI]: 0.64 [0.49, 0.84]) had a lower risk of stroke compared to those with SBP-TTR 0%–25%. Among participants without diabetes, those with SBP-TTR 50%–75% (HR 0.75, 95% CI 0.64–0.88) and 75%–100% (HR [95%CI]: 0.62 [0.52, 0.73]) had a significantly lower risk of stroke. A significant interaction between diabetes status and SBP-TTR was observed (*P* for interaction = 0.03). Additionally, the restricted cubic spline analysis showed a non-linear relationship between SBP-TTR and stroke risk among participants with diabetes (*P* for non-linearity = 0.001), and a linear relationship among those without diabetes (*P* for non-linearity = 0.035).

**Conclusion:**

Higher SBP-TTR was associated with a reduced risk of stroke among participants with or without diabetes. The findings underscore the importance of maintaining blood pressure within the target range to mitigate stroke risk, particularly emphasizing the need for stringent blood pressure control in diabetic patients.

## Introduction

Stroke has become a significant global public health concern. In 2019, the global burden of disease identified stroke as the second leading cause of death worldwide, with 12.2 million new cases (1), including 3.94 million in China (2). Hypertension and diabetes are two major risk factors for stroke, and these two conditions often coexist (3–5). Previous evidence showed that 32% of individuals with hypertension were also diagnosed with diabetes (6), while 60%-75% of patients with diabetes also had hypertension among Chinese population (7). Moreover, the presence of both diabetes and hypertension could significantly increase the risk of stroke (8).

Effective blood pressure (BP) management is fundamental to stroke prevention in people with and without diabetes, with antihypertensive therapy demonstrating significant stroke risk reduction (9). However, BP control targets differ between these groups. For individuals without diabetes, BP management primarily focuses on maintaining systolic blood pressure (SBP) between 120 and 140 mmHg to reduce the risk of stroke (10). However, individuals with diabetes exhibit a significantly higher stroke risk, with women being at greater risk than men, particularly for ischemic stroke (11). Consequently, more intensive BP control is generally recommended for individuals with diabetes, yet the optimal target remains controversial. The Action to Control Cardiovascular Risk in Diabetes (ACCORD) trial found that lowering SBP to <120 mmHg, compared to <140 mmHg, did not reduce the rate of a composite outcome of cardiovascular events in individuals with diabetes (12). In contrast, the Blood Pressure Control Target in Diabetes (BPROAD) trial demonstrated that intensive SBP control (<120 mmHg) significantly lowered the risk of major cardiovascular events (13). These findings not only highlight the ongoing debate over optimal BP management in diabetes but also emphasize the need to paying attention to other BP control measures beyond absolute SBP targets.

Additionally, SBP time in target range (SBP-TTR) is a new metric for evaluating blood pressure control, which refers to the proportion of time that SBP remains within the target range. This metric includes both the average BP value and BP variability over a long-term follow-up period, reflecting BP variation over time (14). According to the Systolic Blood Pressure Intervention Trial (SPRINT) study, higher SBP-TTR was associated with a reduced risk of stroke in a dose-dependent manner (15). Similarly, the ACCORD study found that in individuals with diabetes, higher SBP-TTR was also associated with a lower risk of stroke (16). However, both the SPRINT and ACCORD studies were conducted as randomized controlled trials with specific inclusion criteria, which do not fully capture the complexity and variability of real-world clinical settings, where populations are more diverse and management practices differ. Moreover, there remains a gap in understanding whether this benefit varies between participants with diabetes and without diabetes over longer follow-up periods in real-world settings.

To address these gaps, this study aims to investigate the impact of SBP-TTR on the risk of stroke in people with and without diabetes leveraging data from the Kailuan Study, a large, long-term Chinese cohort.

## Methods

### Study population

The Kailuan Study is a prospective cohort study conducted in Tangshan, China, based on a community population. Detailed descriptions of the study design have been provided in previous articles (17, 18). Since June 2006, the Kailuan General Hospital and its 10 affiliated hospitals have conducted biennial health examination for both current and retired staff members of the Kailuan Group, a large state-owned coal and energy enterprise. This comprehensive assessment was followed by a series of biennial health check-ups and questionnaire-based surveys. This study was approved by the Medical Ethics Committee of Kailuan General Hospital (No. 2018ZX10715005) and adhered strictly to the principles outlined in the Helsinki Declaration. Informed consent was obtained from all participants.

**Figure 1:**
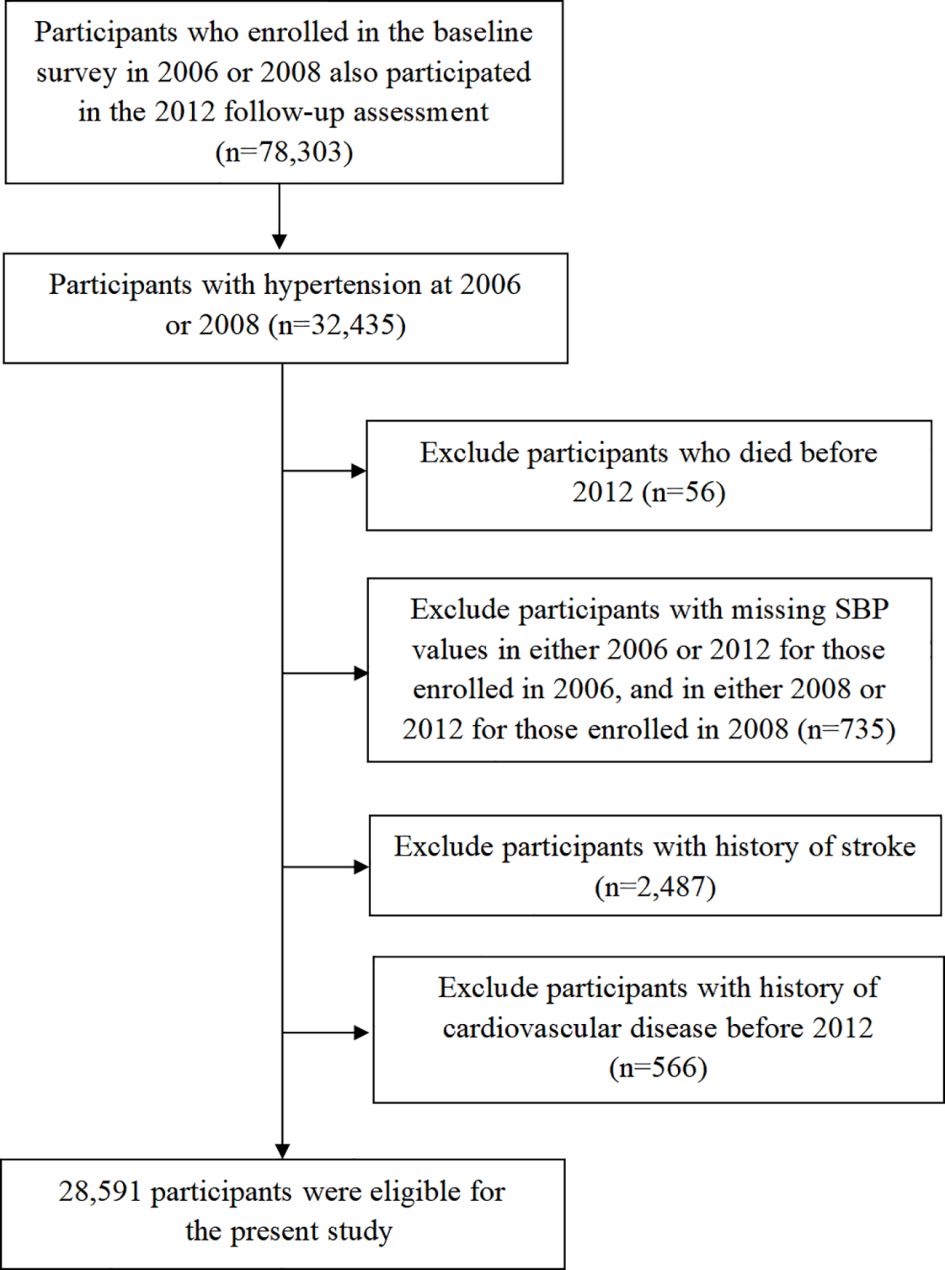
Flow chart of study participants.

Individuals who underwent the Kailuan health examination for the first time in 2006 or 2008, participated in the 2012 follow-up, and were diagnosed with hypertension at their first examination were included in the present analysis. The observation window for participants enrolled in 2006 was from 2006 to 2012, while for those enrolled in 2008, it was from 2008 to 2012. The exclusion criteria were as follows: (1) participants who died before 2012, (2) participants with missing SBP values in either 2006 or 2012 for those enrolled in 2006, and in either 2008 or 2012 for those enrolled in 2008, (3) participants with history of stroke before 2012, and (4) participants with history of cardiovascular disease before 2012 (**Figure 1**).

### Measurement of BP, and definition of SBP-TTR

BP values were measured between 7:00 and 9:00 am on the day of the physical examination using a standard desktop mercury sphygmomanometer. Participants were instructed to sit quietly for at least 5 minutes before the measurement. Three readings were taken at 1 to 2-minute intervals, and the average of these readings was used. In this study, the target range for SBP was set between 120 and 140 mmHg (15). Hypertension was defined as a self-reported history of hypertension, use of antihypertensive medications, SBP ≥140 mmHg, and/or diastolic blood pressure (DBP) ≥90 mmHg. SBP-TTR was defined as the percentage of time that SBP remains within the target range during the observation window. To estimate this, we employed linear interpolation to impute missing SBP values between observation window. For each pair of adjacent time points, a linear model was fitted to estimate the duration within the target range. The time spent in the target range for each interval was then summed to determine the total duration within the target range across the observation window. Finally, SBP-TTR was calculated using the following formula: (time in target range/observation period) *100% (19). SBP-TTR was divided into four groups: 0%<SBP-TTR ≤ 25%, 25% < SBP-TTR ≤ 50%, 50% < SBP-TTR ≤75%, 75% < SBP-TTR ≤ 100%.

### Assessment of stroke

The identification of stroke cases was achieved through meticulous review of medical records (ICD-10 code I60 to I61 for hemorrhagic stroke and I63 for ischemic stroke). All cases were confirmed by professional physicians based on hospitalization records. Participants were followed beginning with the completion of health examinations in 2012 until the occurrence of stroke, death, loss to follow-up, or end of follow-up (December 31, 2020), whichever occurred first.

### Assessment of the covariates

Demographic data (age, sex, income), health behaviors (smoking status, drinking status, education, physical exercise), medical history (diabetes, stroke, cardiovascular disease), and medication use (antihypertensive, lipid-lowering, hypoglycemic drugs) of the participants were collected through questionnaires. Methods for assessing relevant biomarkers (total cholesterol [TC], fasting blood glucose [FBG] and serum creatinine) and anthropometric measurements (height, weight, SBP, DBP) were described in previously published literature (20). Body Mass Index (BMI) was calculated as weight in kilograms divided by the square of height in meters (kg/m²). Current smokers were defined as consuming at least one cigarette per day on average over the past year. Current drinkers were defined as consuming at least 100 mL of liquor (alcohol content ≥ 50%) per day on average over the past year. Education levels were categorized into low (illiterate, primary school, or middle school) and high (high school or college/university). Physically active was defined as engaging in physical activity at least three times per week, with each session lasting at least 30 minutes. Estimated glomerular filtration rate (eGFR) was calculated using the Chronic Kidney Disease Epidemiology Collaboration (CKD-EPI) creatinine equation (21). Urine analysis was conducted using the dry chemical method and urine sediment examination. Urinary protein was measured using the semi-quantitative test strip method. Positive proteinuria was defined as urine dipstick reading equal to or more than 1+. Diabetes was defined as FBG ≥7.0 mmol/L, a self-reported physician diagnosis, or the self-reported use of antidiabetic medications (22, 23). Cardiovascular disease was defined as myocardial infarction, heart failure and atrial fibrillation.

### Statistical analysis

The differences in baseline characteristics by SBP-TTR Group were examined with ANOVA for continuous variables and x^2^ test for categorical variables. The cumulative incidence of endpoint events among different groups was calculated using the Kaplan-Meier method, with inter-group comparisons made using the Log-Rank test. A multivariable-adjusted Cox proportional hazards model was employed to analyze the association of SBP-TTR with the risk of stroke and its subtypes in participants with diabetes and without diabetes. The model adjusted for confounders including age, sex (men, women), smoking status (no, current smokers), drinking status (no, current drinkers), education levels (low, high), physically active (yes, no), BMI, eGFR, TC, baseline SBP, and use of antihypertensive and lipid-lowering medications (yes, no). For participants with diabetes, the models were additionally adjusted for glucose-lowering drugs. We also examined the association between each 10% increase in SBP-TTR and the risk of stroke. Interactions between diabetes status and SBP-TTR on stroke were evaluated including multiplicative interaction terms in the model. Restricted cubic spline models were used to examine the dose-response relationship between SBP-TTR and stroke among individuals with diabetes and without diabetes, with three knots placed at 25th, 50th, and 75th percentiles.

Subgroup analyses were performed based on age (≤60 years, >60 years), sex (men, women), smoking status (no or current smokers), drinking status (no or current drinkers), baseline SBP (<140mmHg, ≥140mmHg) and BMI (<25 kg/m², ≥25 kg/m²). Sensitivity analyses were conducted to ensure the robustness of the results. First, individuals with a follow-up period of 2 years or less were excluded to mitigate the potential reverse causality. Second, the SBP target range was narrowed to 110–130 mmHg. Third, we applied a uniform 6-year SBP-TTR observation window for participants enrolled in 2006 or 2008 to assess the robustness of our findings. Fourth, we further adjusted for proteinuria in the full model. Fifth, given the potential inaccuracies of self-reported data (n=251), diabetes was defined solely based on FBG. Sixth, considering the competing risk of death, Fine-Gray models were used to assess the associations of SBP-TTR and stroke.

All data analyses were conducted using SAS 9.4 (SAS Institute, Cary, North Carolina) and R 4.2 (R Foundation for Statistical Computing) statistical software. In this study, statistical significance was indicated as *p*<0.05 (two-sided test).

## Results

### Baseline characteristics

A total of 28,591 participants were included in the analysis. The majority of participants had an SBP-TTR of 0% to 25% (42.1%), followed by 75% to 100% (24.2%), 25% to 50% (17.3%), and 50% to 75% (16.4%). **Table 1** presents the distribution of baseline characteristics across different SBP-TTR groups among participants with and without diabetes. In general, individuals with higher SBP-TTR were younger, and more likely to be men, current smokers, current drinkers, and higher educated. They also tended to have a lower level of BMI, SBP and DBP, and a lower proportion of receiving antihypertensive drugs treatment.

**Table 1:** Baseline characteristics of individuals with hypertension according to SBP-TTR (n=28,591).

Characteristics	With diabetes (n=6,624)					Without diabetes (n=21,967)				
	>0% to 25%	>25% to 50%	>50% to 75%	>75% to 100%	*P-value*	>0% to 25%	>25% to 50%	>50% to 75%	>75% to 100%	*P-value*
Number of participants	3,226	1,144	957	1,297	–	8,818	3,800	3,724	5,625	–
Age, years	61.3 ± 9.3	58.9 ± 9.5	57.4 ± 9.3	55.1 ± 9.4	<0.01	60.4 ± 10.3	57.0 ± 11.1	55.4 ± 10.7	52.8 ± 10.5	<0.01
Men	2,613 (81.0)	939 (82.2)	799 (83.5)	1,107 (85.4)	<0.01	7,403 (84.0)	3,093 (81.4)	3,098 (83.2)	4,918 (87.4)	<0.01
Current smoker	749 (23.2)	326 (28.5)	281 (29.4)	423 (32.6)	<0.01	2,260 (25.6)	1,040 (27.4)	1,088 (29.2)	1,773 (31.5)	<0.01
Current drinker	828 (25.7)	329 (28.8)	281 (29.4)	415 (32.0)	<0.01	2,423 (27.5)	1,066 (28.1)	1,128 (30.3)	1,961 (34.9)	<0.01
Senior high school or above	330 (10.2)	143 (12.5)	166 (17.3)	266 (20.5)	<0.01	1,102 (12.5)	681 (17.9)	752 (20.2)	1,463 (26.0)	<0.01
Physically active	380 (11.8)	135 (11.8)	104 (10.9)	143 (11.0)	0.80	991 (11.2)	399 (10.5)	360 (9.67)	521 (9.26)	<0.01
BMI, kg/m^2^	26.4 ± 3.5	26.0 ± 3.3	26.1 ± 3.3	26.0 ± 3.5	<0.01	25.7 ± 3.3	25.4 ± 3.4	25.3 ± 3.2	25.5 ± 3.2	<0.01
SBP, mmHg	155.7 ± 17.8	145.5 ± 16.6	142.4 ± 14.9	136.5 ± 10.3	<0.01	152.9 ± 17.5	143.7 ± 15.5	141.0 ± 13.5	135.4 ± 9.9	<0.01
DBP, mmHg	95.0 ± 11.6	92.2 ± 10.7	91.5 ± 9.4	90.5 ± 7.6	<0.01	94.8 ± 10.9	92.4 ± 9.3	91.6 ± 8.7	90.7 ± 7.0	<0.01
FBG, mmol/L	8.4 ± 3.4	8.3 ± 4.0	8.2 ± 3.5	8.3 ± 3.5	0.33	5.4 ± 0.7	5.4 ± 0.7	5.3 ± 0.7	5.3 ± 0.7	<0.01
eGFR, ml/min/1.73 m^2^	72.7 ± 24.8	73.4 ± 26.8	73.9 ± 25.3	76.3 ± 24.2	<0.01	71.8 ± 22.8	73.9 ± 23.1	74.3 ± 23.4	75.5 ± 25.0	<0.01
TC (mmol/L)	5.3 ± 1.5	5.3 ± 2.4	5.2 ± 1.1	5.2 ± 1.2	0.07	5.2 ± 1.5	5.1 ± 1.5	5.1 ± 1.8	5.0 ± 1.3	<0.01
Use of antihypertensive drugs (%)	2,101 (65.1)	593 (51.8)	422 (44.1)	448 (34.5)	<0.01	4,680 (53.1)	1,604 (42.2)	1,221 (32.8)	1,404 (25.0)	<0.01
Use of lipid-lowering drugs (%)	147 (4.6)	50 (4.4)	33 (3.5)	56 (4.3)	0.53	165 (1.9)	76 (2.0)	97 (2.6)	98 (1.7)	0.02
Use of hypoglycemic drugs (%)	1,204 (37.3)	458 (40.0)	315 (32.9)	463 (35.7)	0.02	–	–	–	–	–

Data are present as mean ± standard deviation for continuous variables and N (%) for categorical variables. The differences in baseline characteristics by SBP-TTR Group were examined with ANOVA for continuous variables and x2 test for categorical variables.

BMI, body mass index; SBP, systolic blood pressure; DBP, diastolic blood pressure; eGFR, estimated glomerular filtration rate; FBG, fasting blood glucose; TC, total cholesterol; TTR, time in target range.

Among our study population, 6,624 individuals had diabetes with an average age of 59.1 ± 9.7 years, of whom 5,458 (82.4%) were men. The mean number of BP measurements was 3.4 times. The remaining 21,967 participants were without diabetes, with an average age of 57.0 ± 11.0 years, and 18,512 (84.3%) were men. Compared to participants without diabetes, those with diabetes tended to be older and were more likely to be women. They also had lower education levels, higher BMI and FBG levels. Additionally, a higher percentage of participants with diabetes were receiving antihypertensive and lipid-lowering drug treatments.

### Association between SBP-TTR and stroke

During the mean ± SD follow-up duration of 8.7 ± 1.8 years, we documented 2,206 incident stroke events (2,003 ischemic stroke, and 275 hemorrhagic stroke). The stroke incidence rates per 1,000 person-years across the SBP-TTR categories of 0% to 25%, 25% to 50%, 50% to 75%, and 75% to 100% were 15.35, 14.02, 11.86 and 7.64 for participants with diabetes, and 11.97, 8.37, 6.58, and 4.83, for participants without diabetes (**Table 2**). **Supplementary Figure S1** shows significant differences in the cumulative incidence of stroke among participants with or without diabetes (log-rank test, *P*< 0.001).

**Table 2:** HR (95% CI) of stroke according to SBP-TTR in individuals with or without diabetes (n=28,591).

Variable		TTR Group				*P* for trend	Per 10-point
		>0% to 25%	>25% to 50%	>50% to 75%	>75% to 100%		
Stroke							
Diabetes	Events/N	379/3226	124/1144	91/957	82/1297	–	–
	Incidence rate (per 1000 PYs)	15.35	14.02	11.86	7.64	–	–
	Model	1.00 (reference)	1.04 (0.84, 1.29)	0.92 (0.72, 1.17)	0.64 (0.49, 0.84)	0.003	0.96 (0.94, 0.99)
No diabetes	Events/N	836/8818	260/3800	204/3724	230/5625	–	–
	Incidence rate (per 1000 PYs)	11.97	8.37	6.58	4.83	–	–
	Model	1.00 (reference)	0.87 (0.76, 1.01)	0.75 (0.64, 0.88)	0.62 (0.52, 0.73)	<0.001	0.95 (0.93, 0.96)
*P* interaction		0.03					
Ischemic stroke							
Diabetes	Events/N	350/3226	110/1144	77/957	78/1297	–	–
	Incidence rate (per 1000 PYs)	14.11	12.36	9.97	7.26	–	–
	Model	1.00 (reference)	0.99 (0.80, 1.24)	0.83 (0.64, 1.08)	0.65 (0.50, 0.86)	0.002	0.96 (0.93, 0.98)
No diabetes	Events/N	764/8818	229/3800	189/3724	206/5625	–	–
	Incidence rate (per 1000 PYs)	10.91	7.35	6.08	4.32	–	–
	Model	1.00 (reference)	0.85 (0.73, 0.99)	0.76 (0.64, 0.90)	0.60 (0.51, 0.72)	<0.001	0.95 (0.93, 0.96)
*P* interaction		0.04					
Hemorrhagic stroke							
Diabetes	Events/N	40/3226	15/1144	16/957	4/1297	–	–
	Incidence rate (per 1000 PYs)	1.54	1.62	2.01	0.36	–	–
	Model	1.00 (reference)	1.28 (0.69, 2.39)	1.73 (0.93, 3.22)	0.37 (0.13, 1.11)	0.559	0.98 (0.90, 1.06)
No diabetes	Events/N	102/8818	43/3800	24/3724	31/5625	–	–
	Incidence rate (per 1000 PYs)	1.41	1.35	0.76	0.64	–	–
	Model	1.00 (reference)	1.22 (0.84, 1.77)	0.78 (0.49, 1.24)	0.76 (0.48, 1.19)	0.154	0.97 (0.93, 1.02)
*P* interaction		0.75					

Model was adjusted for age, sex, alcohol drinking status, smoking status, physical activity, education level, BMI, eGFR, TC, SBP, FBG, lipid-lowering and antihypertensive drug use. The models for participants with diabetes were in addition adjusted for glucose-lowering drugs. The *P*-values were generated using the Cox proportional hazards regression models.

BMI, body mass index; SBP, systolic blood pressure; DBP, diastolic blood pressure; eGFR, estimated glomerular filtration rate; FBG, fasting blood glucose; TC, total serum cholesterol; TTR, time in target range.

**Figure 2:**
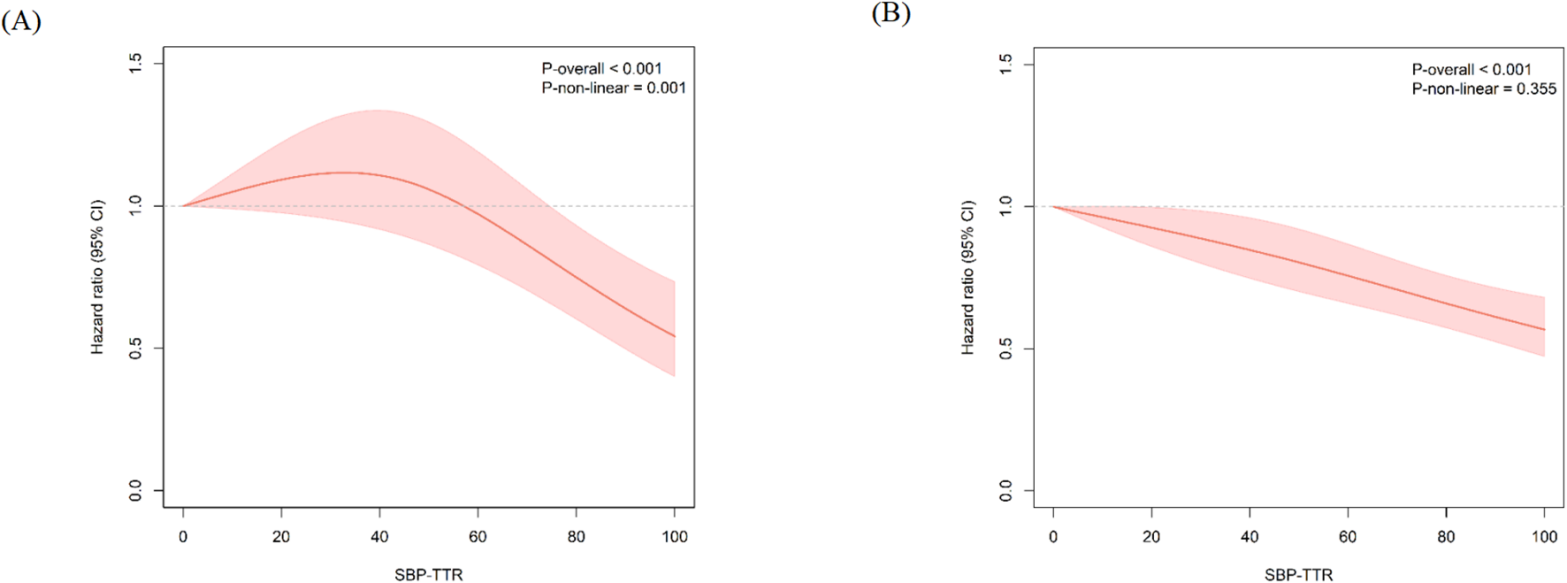
Dose-response association between the SBP-TTR range and risk of stroke using restricted cubic spline. Cox regression models with restricted cubic splines were fitted to the data with SBP-TTR. The solid line represents the point estimate of SBP-TTR correlation with the risk of stroke in individuals with **(A)** or without **(B)** diabetes, and the shaded part represents the 95% CI estimate. Covariates in the model include age, sex, alcohol drinking status, smoking status, physical activity, education level, BMI, eGFR, TC, SBP, FBG, lipid-lowering and antihypertensive drug use. The models for participants with diabetes were in addition adjusted for glucose-lowering drugs. BMI, body mass index; SBP, systolic blood pressure; DBP, diastolic blood pressure; eGFR, estimated glomerular filtration rate; FBG, fasting blood glucose; TC, total serum cholesterol; TTR, time in target range.

Among individuals with diabetes, after adjusting for potential confounders, compared to those with 0%<SBP-TTR ≤ 25%, HRs (95% CIs) for stroke were 1.04 (0.84, 1.29) for 25%<SBP-TTR ≤ 50%, 0.92 (0.72, 1.17) for 50%<SBP-TTR ≤ 75%, and 0.64 (0.49, 0.84) for 75%<SBP-TTR ≤ 100%. In those without diabetes, the HRs (95% CIs) for stroke were 0.87 (0.76, 1.01), 0.75 (0.64, 0.88), and 0.62 (0.52, 0.73), respectively. Moreover, we identified a significant interaction between diabetes and SBP-TTR in the risk of stroke (*P*_for interaction_= 0.03). The relationship between SBP-TTR and stroke risk is non-linear among participants with diabetes (*P*_for non-linearity_ = 0.001), showing maintaining SBP within the target range for a larger percentage of time (beyond 75%) was significantly associated with reduced stroke risk. In contrast, the relationship is linear among participants without diabetes (*P*_for non-linearity_ = 0.355; **Figure 2**). Additionally, the protective effect of increased SBP-TTR was consistent when using ischemic stroke as the outcome (*P*_for interaction_= 0.04). However, no significant association was observed between SBP-TTR and hemorrhagic stroke events in either group (**Table 2**).

### Secondary analyses and subgroup analyses

Stratified analysis showed that the association between SBP-TTR and risk of stroke was more prominent among the younger adults among participants without diabetes (*P* for interaction <0.01). Among individuals with diabetes, SBP-TTR exhibited a stronger protective effect in men (*P* for interaction = 0.05). Additionally, interactions were not significant when stratified by smoking status, alcohol consumption, SBP and BMI in participants with or without diabetes (**Supplementary Table S2**). The robustness of these findings is further supported by the consistency of results when excluding participants with follow-up durations of less than two years. Additionally, strengthening BP reduction to 110–130 mmHg indicated a stronger association between TTR and stroke risk. When applying a uniform 6-year SBP-TTR observation window for participants enrolled in 2006 or 2008, the results remained consistent with the primary findings. After adjusting for proteinuria in the model, using FBG to define diabetes, and using Fine-Gray models, the results remained consistent with the primary findings (**Supplementary Tables S3-S8**).

## Discussion

Using a large cohort study of Chinese, we found that a higher SBP-TTR was associated with a lower risk of stroke in both participants with diabetes and without diabetes, with significant interactions suggesting a stronger protective effect in individuals without diabetes. Similar patterns were found for ischemic stroke but not for hemorrhagic stroke. Additionally, among individuals with diabetes, the protective effect of SBP-TTR was more pronounced in men than in women.

Hypertension stands as the foremost risk factor for stroke (24, 25), with prior studies primarily focusing on evaluating and monitoring the efficacy of BP management through single BP measurements or mean BP values. SBP is influenced by multiple factors and internal regulatory mechanisms, existing within a dynamic equilibrium. Therefore, it is imperative that we adopt a more comprehensive approach to consider the temporal variations in BP. Compared to traditional indicators, SBP-TTR better reflects the dynamic fluctuations of BP, and accounts for changes in BP trends over a period, thereby more accurately assessing the efficacy of BP management. Previous studies have indicated that higher SBP-TTR are associated with lower risk of cardiovascular disease (15, 26, 27). However, whether different SBP-TTR targets are needed for participants with diabetes is limited.

In agreement with previous studies, our data showed that a higher SBP-TTR was associated with a lower risk of stroke in both participants with diabetes and without diabetes, yet none of which has further stratified the analysis based on diabetes status to explore whether the protective effects exhibit any differences. Previous studies have widely recognized BP control as established strategy for reducing the risk of stroke (28). Per 10 mm Hg reduction in SBP significantly reduced the risk of stroke by an estimated 27% (29). The UK Prospective Diabetes Study (UKPDS) results showed that strict BP control in participants with diabetes and hypertension can reduce the risk of stroke by 44% (30). SBP-TTR serves as an indicator for assessing long-term BP management. Buckley et al.’s study results demonstrated a significant relationship between SBP-TTR and cardiovascular risk in individuals with diabetes and without diabetes (31). Additionally, SPRINT study and ACCORD study, which focused on individuals with hypertension alone and those with hypertension and diabetes, respectively, also demonstrated that increased SBP-TTR was associated with a reduced risk of stroke (15, 16).

Moreover, our data further demonstrated that the protective effect of SBP-TTR was stronger in individuals without diabetes. Among individuals with diabetes, SBP-TTR needs to be maintained above 75% to achieve a notable protective effect against stroke. A similar result was found in a meta-analysis of 51 randomized trials, which showed that antihypertensive treatment can reduce cardiovascular risk in participants with diabetes, although the effects were weaker than those without diabetes (32). Of note, the present study implied that a linear association between SBP-TTR and risk of stroke in people without diabetes, consistent with prior studies (15, 31). Conversely, among those with diabetes, this association deviates from linearity. This divergence is potentially attributable to the observation that individuals with diabetes and SBP-TTR exceeding 75% exhibit significantly lower baseline SBP and FBG levels compared to those with SBP-TTR below 75%. Additionally, diabetes may induce comorbidities such as vascular damage and autonomic neuropathy, which present challenges in BP management (33, 34). This study also found a significant protective effect of SBP-TTR in male but not female participants with diabetes. This sex difference may be attributed to greater arterial stiffness, more pronounced microvascular dysfunction, and a higher stroke risk in women with diabetes, which could attenuate the protective impact of BP control (35, 36). Our finding has several important clinical implications. First, it highlights the need for different strategies in BP management for participants with and without diabetes. For patients with diabetes, the goal should be to maintain SBP-TTR above 75% to significantly reduce the risk of stroke. Second, since SBP-TTR reflects long-term BP control and variability, clinicians should focus more on long-term monitoring rather than single BP measurements. This implies that regular BP monitoring and adjustments to treatment plans based on BP trends over time are essential in daily clinical practice. Finally, patients with diabetes should be treated as a high-risk group for more stringent BP control. Clinicians should stratify patients based on their individual risk factors and implement personalized and precise BP management strategies to enhance clinical outcomes.

Diabetes and hypertension frequently coexist, sharing considerable overlap in their etiologies and mechanisms, with a bidirectional pathogenic relationship (5, 37). Hypertension usually occurs before diabetes and the population base of individuals with hypertension is much larger than that of individuals with both hypertension and diabetes (37, 38). Therefore, strategies to improve SBP-TTR levels in individuals with hypertension can not only reduce the risk of diabetes but also lower the risk of stroke, achieving dual health benefits and greater societal impact. Significantly extending SBP-TTR is essential for achieving a marked protective effect against stroke among participants with diabetes. Furthermore, our findings demonstrated that regardless of whether the baseline SBP was <140 mmHg or ≥140 mmHg, higher SBP-TTR levels were associated with lower stroke risk in both groups. Proactive BP management to extend SBP-TTR should be pursued regardless of an individual’s BP levels to reduce the risk of stroke. Additionally, we further categorized stroke into ischemic and hemorrhagic subtypes, observed that SBP-TTR was significantly associated with ischemic stroke but not with hemorrhagic stroke. This may be attributed to the relatively small number of hemorrhagic stroke cases in our study and the fact that SBP-TTR primarily reflects long-term BP control rather than acute fluctuations. Given that hemorrhagic stroke is more closely associated with sudden and extreme BP elevations, this could explain the lack of a significant association. This highlights the necessity of considering the distinct pathologies and underlying mechanisms of different stroke types in public health strategies and clinical practice. Personalized prevention and management approaches should be adopted for different stroke subtypes to achieve optimal cost-effectiveness.

This is among the first large-scale prospective studies investigating the impact of SBP-TTR on stroke in participants with and without diabetes. Other strengths of this study include its large sample size, relatively long follow-up duration, and standardized BP measurements. However, our study has several limitations. First, the study participants were employees of a specific corporation, and predominantly men, which may limit the generalizability of the findings to other populations with different social backgrounds. Second, we diagnosed diabetes using FBG level, self-reported disease and therapy history, without assessing oral glucose tolerance tests or levels of hemoglobin A1c, which may underestimate the incidence of diabetes. Third, our study used office BP measurements to assess SBP-TTR, rather than home or ambulatory BP monitoring, which could introduce bias due to phenomena such as white-coat hypertension or masked hypertension. Fourth, our study did not differentiate between ischemic stroke subtypes, which may have differing underlying mechanisms and risk factor associations. Future research should further explore these subtypes, particularly lacunar infarction, as hypertension and diabetes are its primary risk factors (39). Fifth, A potential limitation of our study is that BP was measured every two years, which may not fully capture short-term fluctuations. However, SBP-TTR was estimated using linear interpolation, consistent with prior studies. Given the large-scale design and extended follow-up period of this real-world study, conducting more frequent BP measurements would have been challenging. Despite this limitation, our findings provide important evidence on the impact of SBP-TTR on stroke risk. Finally, as this study is observational in nature, causality between SBP-TTR and the risk of stroke cannot be established.

In conclusion, the Kailuan study highlights the critical role of SBP-TTR in reducing stroke risk. While stringent BP control is particularly important for individuals with diabetes, our findings also indicate that a higher SBP-TTR is associated with greater protective effects in individuals without diabetes. This underscores the importance of optimal BP control for all individuals, regardless of diabetes status. These results have significant implications for clinical practice, suggesting that tailored BP management strategies that maximize SBP-TTR could reduce the risk of stroke across different risk groups. Future research is required to validate our findings and elucidate the exact mechanisms underlying the association between SBP-TTR and stroke risk in individuals with and without diabetes.

## Data Availability

The original contributions presented in the study are included in the article/**Supplementary Material**. Further inquiries can be directed to the corresponding author/s.
