# Supplementary material for: Time in target range for systolic blood pressure and stroke in people with and without diabetes: the Kailuan prospective cohort study
Source: Front Endocrinol (Lausanne). 2025 May 14;16:1537343. doi: 10.3389/fendo.2025.1537343 (PMC12116358; doi:10.3389/fendo.2025.1537343)
Supplement: Supplementary file 1 [file DataSheet1.docx]

**SUPPLEMENTAL MATERIAL**

**Table S1. Baseline characteristics of overall individuals with hypertension, stratified by diabetic status**

**Table S2. HR (95% CI) of stroke according to SBP-TTR in individuals with or without diabetes, stratified by age, sex, smoking status, SBP and BMI**

**Table S3. HR (95% CI) of stroke according to SBP-TTR in individuals with hypertension after excluding stroke events that occurred within the first two years of follow-up**

**Table S4. HR (95% CI) of stroke according to SBP-TTR in individuals with hypertension using 110-130 mm Hg as the SBP target range**

**Table S5. HR (95% CI) of stroke according to SBP-TTR with a uniform 6-year observation window in individuals with hypertension Table S6. HR (95% CI) of stroke according to SBP-TTR in individuals with or without diabetes as defined by FBG**

**Table S7. HR (95% CI) of stroke according to SBP-TTR in individuals with or without diabetes** **with the adjustment for proteinuria**

**Table S8. HR (95% CI) of stroke according to SBP-TTR in individuals with** hypertension **using the competing risk model**

**Figure S1. Kaplan-Meier curves of cumulative incidence of stroke in individuals with or without diabetes**

| **Table S1.** **Baseline characteristics of overall individuals with hypertension, stratified by diabetic status.** | | | | |
| --- | --- | --- | --- | --- |
| **Characteristics** | Overall | With diabetes | Without diabetes | ***P-value*** |
| Number of participants | 28,591 | 6,624 | 21,967 | - |
| Age, years | 57.5±10.7 | 59.1±9.7 | 57.0±11.0 | <0.01 |
| Men | 23,970 (83.8) | 5,458 (82.4) | 18512 (84.3) | <0.01 |
| Current smoker | 7,940 (27.8) | 1,779 (26.9) | 6,161 (28.0) | 0.06 |
| Current drinker | 8,431 (29.5) | 1,853 (28.0) | 6,578 (29.9) | <0.01 |
| Senior high school or above | 4,903 (17.1) | 905 (13.7) | 3,998 (18.2) | <0.01 |
| Physically active | 3,033 (10.6) | 762 (11.5) | 2,271 (10.3) | <0.01 |
| BMI, kg/m^2^ | 25.7±3.3 | 26.2±3.4 | 25.5±3.3 | <0.01 |
| SBP, mmHg | 145.6±16.9 | 148.3±17.8 | 144.8±16.5 | <0.01 |
| DBP, mmHg | 92.9±9.8 | 93.2±10.7 | 92.8±9.5 | 0.24 |
| FBG, mmol/L | 6.1±2.2 | 8.3±3.6 | 5.4±0.7 | <0.01 |
| eGFR, ml/min/1.73 m2 | 73.6±24.0 | 73.7±25.1 | 73.6±23.6 | 0.65 |
| TC (mmol/L) | 5.1±1.5 | 5.3±1.6 | 5.1±1.5 | <0.01 |
| Use of antihypertensive drugs (%) | 12,473 (43.6) | 3,564 (53.8) | 8,909 (40.6) | <0.01 |
| Use of lipid-lowering drugs (%) | 722 (2.5) | 286 (4.3) | 436 (2.0) | <0.01 |
| Use of hypoglycemic drugs (%) | 2,440 (8.5) | 2,440 (36.8) | - | <0.01 |
| Data are present as mean ± standard deviation for continuous variables and N (%) for categorical variables.  Abbreviations: BMI, body mass index; SBP, systolic blood pressure; DBP, diastolic blood pressure; eGFR, estimated glomerular filtration rate; FBG, fasting blood glucose; TC, total cholesterol. | | | | |

| **Table S2. HR (95% CI) of stroke according to SBP-TTR in** individuals with or without diabetes, stratified by age, sex, smoking status, SBP and BMI. | | | | | | |
| --- | --- | --- | --- | --- | --- | --- |
|  | **TTR Group** | | | | ***P* for trend** | **Per 10-point** |
|  | **>0% to 25%** | **>25% to 50%** | **>50% to 75%** | **>75% to 100%** |  |  |
| **Age, years** |  |  |  |  |  |  |
| Diabetes |  |  |  |  |  |  |
| <65 (n=4,950) | 1.00 (reference) | 1.05 (0.82, 1.34) | 1.00 (0.76, 1.32) | 0.65 (0.48, 0.88) | 0.017 | 0.96 (0.93, 0.99) |
| ≥65 (n=1,674) | 1.00 (reference) | 1.03 (0.69, 1.55) | 0.68 (0.40, 1.17) | 0.72 (0.40, 1.32) | 0.146 | 0.96 (0.91, 1.02) |
| ***P* interaction** | 0.21 |  |  |  |  |  |
| No diabetes |  |  |  |  |  |  |
| <65 (n=17,282) | 1.00 (reference) | 0.80 (0.67, 0.96) | 0.74 (0.62, 0.90) | 0.60 (0.50, 0.73) | <0.001 | 0.94 (0.92, 0.96) |
| ≥65 (n=4,685) | 1.00 (reference) | 1.02 (0.80, 1.30) | 0.72 (0.53, 0.98) | 0.72 (0.52, 1.00) | 0.017 | 0.96 (0.93, 1.00) |
| ***P* interaction** | **<0.01** |  |  |  |  |  |
| **Sex** |  |  |  |  |  |  |
| Diabetes |  |  |  |  |  |  |
| Men (n=5,458) | 1.00 (reference) | 1.06 (0.85, 1.33) | 0.87 (0.67, 1.13) | 0.57 (0.43, 0.77) | <0.001 | 0.95 (0.93, 0.98) |
| Women (n=1,165) | 1.00 (reference) | 0.99 (0.53, 1.83) | 1.30 (0.69, 2.45) | 1.27 (0.66, 2.45) | 0.370 | 1.01 (0.94, 1.09) |
| ***P* interaction** | 0.05 |  |  |  |  |  |
| No diabetes |  |  |  |  |  |  |
| Men (n=18,512) | 1.00 (reference) | 0.84 (0.72, 0.98) | 0.72 (0.61, 0.86) | 0.59 (0.49, 0.70) | <0.001 | 0.94 (0.93, 0.96) |
| Women (n=3,452) | 1.00 (reference) | 1.20 (0.81, 1.78) | 0.95 (0.59, 1.50) | 0.96 (0.58, 1.56) | 0.798 | 0.99 (0.94, 1.04) |
| ***P* interaction** | 0.19 |  |  |  |  |  |
| **Smoking status** |  |  |  |  |  |  |
| Diabetes |  |  |  |  |  |  |
| Current smoker (n=1,179) | 1.00 (reference) | 1.06 (0.72, 1.55) | 1.11 (0.74, 1.66) | 0.63 (0.39, 1.01) | 0.153 | 0.96 (0.92, 1.01) |
| Non-current smoker (n=4,845) | 1.00 (reference) | 1.05 (0.81, 1.35) | 0.85 (0.63, 1.14) | 0.67 (0.49, 0.93) | 0.018 | 0.96 (0.93, 0.99) |
| ***P* interaction** | 0.56 |  |  |  |  |  |
| No diabetes |  |  |  |  |  |  |
| Current smoker (n=6,161) | 1.00 (reference) | 0.82 (0.64, 1.06) | 0.72 (0.54, 0.95) | 0.56 (0.42, 0.75) | <0.001 | 0.93 (0.91, 0.96) |
| Non-current smoker (n=15,806) | 1.00 (reference) | 0.90 (0.76, 1.07) | 0.77 (0.63, 0.94) | 0.66 (0.54, 0.80) | <0.001 | 0.96 (0.94, 0.98) |
| ***P* interaction** | 0.06 |  |  |  |  |  |
| **Alcohol intake** |  |  |  |  |  |  |
| Diabetes |  |  |  |  |  |  |
| Current drinker (n=1853) | 1.00 (reference) | 0.86 (0.57, 1.28) | 0.71 (0.44, 1.14) | 0.43 (0.25, 0.73) | 0.002 | 0.92 (0.88, 0.97) |
| Non-current drinker (n=4771) | 1.00 (reference) | 1.13 (0.88, 1.45) | 1.03 (0.77, 1.36) | 0.76 (0.56, 1.04) | 0.175 | 0.98 (0.95, 1.01) |
| ***P* interaction** | 0.08 |  |  |  |  |  |
| No diabetes |  |  |  |  |  |  |
| Current drinker (n=6578) | 1.00 (reference) | 0.77 (0.58, 1.01) | 0.70 (0.52, 0.94) | 0.60 (0.45, 0.80) | <0.001 | 0.94 (0.91, 0.97) |
| Non-current drinker (n=15,389) | 1.00 (reference) | 0.93 (0.78, 1.10) | 0.78 (0.64, 0.94) | 0.63 (0.52, 0.77) | <0.001 | 0.95 (0.93, 0.97) |
| ***P* interaction** | 0.25 |  |  |  |  |  |
| **SBP, mmHg** |  |  |  |  |  |  |
| Diabetes |  |  |  |  |  |  |
| <140 (n=1,571) | 1.00 (reference) | 0.66 (0.39, 1.11) | 0.72 (0.42, 1.22) | 0.39 (0.23, 0.65) | <0.001 | 0.90 (0.85, 0.95) |
| ≥140 (n=5,053) | 1.00 (reference) | 1.13 (0.90, 1.42) | 0.93 (0.71, 1.23) | 0.77 (0.56, 1.05) | 0.164 | 0.98 (0.95, 1.01) |
| ***P* interaction** | 0.05 |  |  |  |  |  |
| No diabetes |  |  |  |  |  |  |
| <140 (n=6,587) | 1.00 (reference) | 1.08 (0.76, 1.54) | 0.90 (0.63, 1.30) | 0.66 (0.47, 0.92) | 0.003 | 0.95 (0.92, 0.98) |
| ≥140 (n=15,380) | 1.00 (reference) | 0.83 (0.70, 0.97) | 0.70 (0.58, 0.84) | 0.65 (0.54, 0.80) | <0.001 | 0.95 (0.93, 0.97) |
| ***P* interaction** | 0.19 |  |  |  |  |  |
| **BMI kg/m^2^** |  |  |  |  |  |  |
| Diabetes |  |  |  |  |  |  |
| <28 (n=4,930) | 1.00 (reference) | 1.02 (0.80, 1.30) | 0.95 (0.72, 1.24) | 0.58 (0.43, 0.79) | 0.003 | 0.95 (0.92, 0.98) |
| ≥28 (n=1,694) | 1.00 (reference) | 1.07 (0.69, 1.65) | 0.80 (0.47, 1.36) | 0.84 (0.50, 1.41) | 0.387 | 0.99 (0.94, 1.04) |
| ***P* interaction** | 0.29 |  |  |  |  |  |
| No diabetes |  |  |  |  |  |  |
| <28 (n=17,628) | 1.00 (reference) | 0.86 (0.73, 1.01) | 0.71 (0.59, 0.85) | 0.62 (0.52, 0.75) | <0.001 | 0.95 (0.93, 0.96) |
| ≥28 (n=4,339) | 1.00 (reference) | 0.92 (0.67, 1.26) | 0.96 (0.68, 1.36) | 0.61 (0.42, 0.89) | 0.028 | 0.96 (0.92, 0.99) |
| ***P* interaction** | 0.19 |  |  |  |  |  |
| Model was adjusted for age, sex, alcohol drinking status, smoking status, physical activity, education level, BMI, eGFR, TC, SBP, FBG, lipid-lowering and antihypertensive drug use. The models for participants with diabetes were in addition adjusted for glucose-lowering drugs. | | | | | | |
| Abbreviations: BMI, body mass index; SBP, systolic blood pressure; DBP, diastolic blood pressure; eGFR, estimated glomerular filtration rate; FBG, fasting blood glucose; TC, total serum cholesterol; TTR, time in target range. | | | | | | |

| **Table S3. HR (95% CI) of stroke according to SBP-TTR in individuals with hypertension after excluding stroke events that occurred within the first two years of follow-up.** | | | | | | | |
| --- | --- | --- | --- | --- | --- | --- | --- |
|  | | **TTR Group** | | | | ***P* for trend** | **Per 10-point** |
|  |  | **>0% to 25%** | **>25% to 50%** | **>50% to 75%** | **>75% to 100%** |  |  |
| **Stroke** | |  |  |  |  |  |  |
| Diabetes | Events/N | 304/3151 | 92/1112 | 76/942 | 69/1284 | - | - |
|  | Incidence rate | 12.35 | 10.44 | 9.92 | 6.44 | - | - |
|  | HR (95% CI) | 1.00 (reference) | 0.93 (0.73,1.19) | 0.91 (0.70, 1.19) | 0.63 (0.47, 0.84) | 0.004 | 0.96(0.93,0.99) |
| No diabetes | Events/N | 693/8675 | 219/3759 | 171/3691 | 201/5596 | - | - |
|  | Incidence rate | 9.95 | 7.06 | 5.52 | 4.22 | - | - |
|  | HR (95% CI) | 1.00 (reference) | 0.88 (0.75, 1.03) | 0.74 (0.62, 0.89) | 0.64 (0.53, 0.76) | <0.001 | 0.95(0.93,0.97) |
| **Ischemic stroke** | |  |  |  |  |  |  |
| Diabetes | Events/N | 285/3161 | 85/1119 | 65/945 | 66/1285 | - | - |
|  | Incidence rate | 11.52 | 9.58 | 8.43 | 6.16 | - | - |
|  | HR (95% CI) | 1.00 (reference) | 0.91 (0.71, 1.17) | 0.82 (0.62, 1.09) | 0.63 (0.47, 0.85) | 0.003 | 0.95 (0.93, 0.98) |
| No diabetes | Events/N | 639/8693 | 198/3769 | 160/3695 | 183/5602 | - | - |
|  | Incidence rate | 9.14 | 6.36 | 5.15 | 3.84 | - | - |
|  | HR (95% CI) | 1.00 (reference) | 0.86 (0.73, 1.02) | 0.76 (0.63, 0.91) | 0.63 (0.52, 0.76) | <0.001 | 0.95 (0.93, 0.97) |
| **Hemorrhagic stroke** | |  |  |  |  |  |  |
| Diabetes | Events/N | 27/3213 | 8/1137 | 12/953 | 3/1296 | - | - |
|  | Incidence rate | 1.04 | 0.86 | 1.51 | 0.27 | - | - |
|  | HR (95% CI) | 1.00 (reference) | 1.05 (0.46, 2.39) | 2.05 (0.98, 4.30) | 0.46 (0.13, 1.63) | 0.968 | 1.00 (0.91, 1.10) |
| No diabetes | Events/N | 80/8796 | 32/3789 | 20/3720 | 25/5619 | - | - |
|  | Incidence rate | 1.10 | 1.01 | 0.63 | 0.52 | - | - |
|  | HR (95% CI) | 1.00 (reference) | 1.21 (0.79, 1.85) | 0.84 (0.50, 1.40) | 0.79 (0.48, 1.32) | 0.304 | 0.98 (0.93, 1.03) |
| The incidence rate is per 1000 person-years  Model was adjusted for age, sex, alcohol drinking status, smoking status, physical activity, education level, BMI, eGFR, TC, SBP, FBG, lipid-lowering and antihypertensive drug use. The models for participants with diabetes were in addition adjusted for glucose-lowering drugs. | | | | | | | |
| Abbreviations: BMI, body mass index; SBP, systolic blood pressure; DBP, diastolic blood pressure; eGFR, estimated glomerular filtration rate; FBG, fasting blood glucose; TC, total serum cholesterol; TTR, time in target range. | | | | | | | |

| **Table S4. HR (95% CI) of stroke according to SBP-TTR in individuals with** hypertension **using 110-130 mm Hg as the SBP target range. (n=**28,591**)** | | | | | | | |
| --- | --- | --- | --- | --- | --- | --- | --- |
|  | | **TTR Group** | | | | ***P* for trend** | **Per 10-point** |
|  |  | **>0% to 25%** | **>25% to 50%** | **>50% to 75%** | **>75% to 100%** |  |  |
| **Stroke** | |  |  |  |  |  |  |
| Diabetes | Events/N | 550/4912 | 63/776 | 33/481 | 30/455 | - | - |
|  | Incidence rate | 14.47 | 10.18 | 8.29 | 7.99 | - | - |
|  | HR (95% CI) | 1.00 (reference) | 0.80 (0.61, 1.05) | 0.78 (0.53, 1.14) | 0.68 (0.48, 0.98) | 0.018 | 0.96 (0.93, 0.99) |
| No diabetes | Events/N | 1227/14317 | 142/2887 | 95/2197 | 66/2566 | - | - |
|  | Incidence rate | 10.67 | 5.93 | 5.12 | 3.00 | - | - |
|  | HR (95% CI) | 1.00 (reference) | 0.71 (0.60, 0.85) | 0.69 (0.55, 0.85) | 0.47 (0.36, 0.61) | <0.001 | 0.92 (0.90, 0.95) |
| ***P* interaction** | | <0.01 |  |  |  |  |  |
| **Ischemic stroke** | |  |  |  |  |  |  |
| Diabetes | Events/N | 502/4912 | 56/776 | 30/481 | 27/455 | - | - |
|  | Incidence rate | 13.14 | 9.01 | 7.53 | 7.15 | - | - |
|  | HR (95% CI) | 1.00 (reference) | 0.78 (0.59, 1.45) | 0.77 (0.51, 1.15) | 0.68 (0.47, 0.99) | 0.019 | 0.95 (0.92, 0.99) |
| No diabetes | Events/N | 1113/14317 | 125/2887 | 88/2197 | 62/2566 | - | - |
|  | Incidence rate | 9.65 | 5.20 | 4.74 | 2.82 | - | - |
|  | HR (95% CI) | 1.00 (reference) | 0.70 (0.58, 0.84) | 0.70 (0.57, 0.89) | 0.49 (0.38 0.65) | <0.001 | 0.93 (0.91, 0.95) |
| ***P* interaction** | | 0.02 |  |  |  |  |  |
| **Hemorrhagic stroke** | |  |  |  |  |  |  |
| Diabetes | Events/N | 60/4912 | 8/776 | 3/481 | 4/455 | - | - |
|  | Incidence rate | 1.51 | 1.25 | 0.73 | 1.04 | - | - |
|  | HR (95% CI) | 1.00 (reference) | 1.03 (0.48, 2.20) | 0.64 (0.19, 2.09) | 1.14 (0.39, 3.34) | 0.852 | 1.02 (0.92, 1.12) |
| No diabetes | Events/N | 162/14317 | 20/2887 | 12/2197 | 6/2566 | - | - |
|  | Incidence rate | 1.36 | 0.82 | 0.64 | 0.27 | - | - |
|  | HR (95% CI) | 1.00 (reference) | 0.78 (0.48, 1.25) | 0.65 (0.35, 1.19) | 0.32 (0.14, 0.76) | 0.004 | 0.91 (0.85, 0.97) |
| ***P* interaction** | | 0.04 |  |  |  |  |  |
| The incidence rate is per 1000 person-years  Model was adjusted for age, sex, alcohol drinking status, smoking status, physical activity, education level, BMI, eGFR, TC, SBP, FBG, lipid-lowering and antihypertensive drug use. The models for participants with diabetes were in addition adjusted for glucose-lowering drugs. | | | | | | | |
| Abbreviations: BMI, body mass index; SBP, systolic blood pressure; DBP, diastolic blood pressure; eGFR, estimated glomerular filtration rate; FBG, fasting blood glucose; TC, total serum cholesterol; TTR, time in target range. | | | | | | | |

| **Table S5. HR (95% CI) of stroke according to SBP-TTR with a uniform 6-year observation window in individuals with hypertension. (n=**27,096**)** | | | | | | | |
| --- | --- | --- | --- | --- | --- | --- | --- |
|  | | **TTR Group** | | | | ***P* for trend** | **Per 10-point** |
|  |  | **>0% to 25%** | **>25% to 50%** | **>50% to 75%** | **>75% to 100%** |  |  |
| **Stroke** | |  |  |  |  |  |  |
| Diabetes | Events/N | 355/3050 | 126/1186 | 85/990 | 78/1211 | - | - |
|  | Incidence rate | 8.58 | 7.73 | 6.12 | 4.54 | - | - |
|  | HR (95% CI) | 1.00 (reference) | 1.04 (0.85, 1.29) | 0.85 (0.66, 1.09) | 0.68 (0.51, 0.89) | 0.004 | 0.96 (0.94, 0.99) |
| No diabetes | Events/N | 755/8141 | 258/3735 | 188/3708 | 207/5075 | - | - |
|  | Incidence rate | 6.62 | 4.87 | 3.54 | 2.84 | - | - |
|  | HR (95% CI) | 1.00 (reference) | 0.91 (0.79, 1.05) | 0.73 (0.61, 0.86) | 0.64 (0.54, 0.76) | <0.001 | 0.95 (0.93, 0.97) |
| ***P* interaction** | | 0.02 |  |  |  |  |  |
| **Ischemic stroke** | |  |  |  |  |  |  |
| Diabetes | Events/N | 327/3050 | 114/1186 | 72/990 | 74/1211 | - | - |
|  | Incidence rate | 7.78 | 6.98 | 5.17 | 4.30 | - | - |
|  | HR (95% CI) | 1.00 (reference) | 1.02 (0.82, 1.27) | 0.77 (0.59, 1.01) | 0.69 (0.52, 0.91) | 0.004 | 0.96 (0.93, 0.99) |
| No diabetes | Events/N | 692/8141 | 226/3735 | 177/3708 | 185/5075 | - | - |
|  | Incidence rate | 6.06 | 4.26 | 3.33 | 2.53 | - | - |
|  | HR (95% CI) | 1.00 (reference) | 0.87 (0.74, 1.01) | 0.74 (0.62, 0.89) | 0.62 (0.52, 0.75) | <0.001 | 0.95 (0.93, 0.96) |
| ***P* interaction** | | 0.03 |  |  |  |  |  |
| **Hemorrhagic stroke** | |  |  |  |  |  |  |
| Diabetes | Events/N | 39/3050 | 13/1186 | 15/990 | 5/1211 | - | - |
|  | Incidence rate | 0.91 | 0.78 | 1.06 | 0.29 | - | - |
|  | HR (95% CI) | 1.00 (reference) | 1.07 (0.56, 2.06) | 1.57 (0.82, 2.98) | 0.49 (0.18, 1.33) | 0.653 | 0.98 (0.91, 1.07) |
| No diabetes | Events/N | 94/8141 | 41/3735 | 18/3708 | 27/5075 | - | - |
|  | Incidence rate | 0.81 | 0.76 | 0.34 | 0.37 | - | - |
|  | HR (95% CI) | 1.00 (reference) | 1.23 (0.84, 1.81) | 0.61 (0.36, 1.03) | 0.75 (0.46, 1.21) | 0.097 | 0.97 (0.92, 1.01) |
| ***P* interaction** | | 0.71 |  |  |  |  |  |
| The incidence rate is per 1000 person-years  Model was adjusted for age, sex, alcohol drinking status, smoking status, physical activity, education level, BMI, eGFR, TC, SBP, FBG, lipid-lowering and antihypertensive drug use. The models for participants with diabetes were in addition adjusted for glucose-lowering drugs. | | | | | | | |
| Abbreviations: BMI, body mass index; SBP, systolic blood pressure; DBP, diastolic blood pressure; eGFR, estimated glomerular filtration rate; FBG, fasting blood glucose; TC, total serum cholesterol; TTR, time in target range. | | | | | | | |

| **Table S6. HR (95% CI) of stroke according to SBP-TTR in individuals with or without diabetes as defined by FBG. (n=28,340)** | | | | | | | |
| --- | --- | --- | --- | --- | --- | --- | --- |
|  | | **TTR Group** | | | | ***P* for trend** | **Per 10-point** |
|  |  | **>0% to 25%** | **>25% to 50%** | **>50% to 75%** | **>75% to 100%** |  |  |
| **Stroke** | |  |  |  |  |  |  |
| Diabetes | Events/N | 369/3106 | 118/1102 | 88/925 | 78/1240 | - | - |
|  | Incidence rate (per 1000 PYs) | 15.55 | 11.52 | 11.87 | 7.60 | - | - |
|  | Model | 1.00 (reference) | 1.01 (0.81, 1.25) | 0.90 (0.70, 1.15) | 0.62 (0.48, 0.82) | 0.002 | 0.96 (0.93, 0.98) |
| No diabetes | Events/N | 836/8818 | 260/3800 | 204/3724 | 230/5625 | - | - |
|  | Incidence rate (per 1000 PYs) | 11.97 | 8.37 | 6.58 | 4.83 | - | - |
|  | Model | 1.00 (reference) | 0.87 (0.76, 1.01) | 0.75 (0.64, 0.88) | 0.62 (0.52, 0.73) | <0.001 | 0.95 (0.93, 0.96) |
| ***P* interaction** | | 0.03 |  |  |  |  |  |
| **Ischemic stroke** | |  |  |  |  |  |  |
| Diabetes | Events/N | 343/3106 | 105/1102 | 74/925 | 74/1240 | - | - |
|  | Incidence rate (per 1000 PYs) | 14.40 | 12.21 | 9.91 | 7.21 | - | - |
|  | Model | 1.00 (reference) | 0.96 (0.77, 1.21) | 0.81 (0.62, 1.05) | 0.63 (0.48, 0.84) | 0.001 | 0.95 (0.93, 0.98) |
| No diabetes | Events/N | 764/8818 | 229/3800 | 189/3724 | 206/5625 | - | - |
|  | Incidence rate (per 1000 PYs) | 10.91 | 7.35 | 6.08 | 4.32 | - | - |
|  | Model | 1.00 (reference) | 0.85 (0.73, 0.99) | 0.76 (0.64, 0.90) | 0.60 (0.51, 0.72) | <0.001 | 0.95 (0.93, 0.96) |
| ***P* interaction** | | 0.06 |  |  |  |  |  |
| **Hemorrhagic stroke** | |  |  |  |  |  |  |
| Diabetes | Events/N | 36/3106 | 14/1102 | 16/925 | 4/1240 | - | - |
|  | Incidence rate (per 1000 PYs) | 1.44 | 1.56 | 2.08 | 0.38 | - | - |
|  | Model | 1.00 (reference) | 1.31 (0.69, 2.50) | 1.88 (0.99, 3.56) | 0.41 (0.14, 1.22) | 0.730 | 0.98 (0.91, 1.07) |
| No diabetes | Events/N | 102/8818 | 43/3800 | 24/3724 | 31/5625 | - | - |
|  | Incidence rate (per 1000 PYs) | 1.41 | 1.35 | 0.76 | 0.64 | - | - |
|  | Model | 1.00 (reference) | 1.22 (0.84, 1.77) | 0.78 (0.49, 1.24) | 0.76 (0.48, 1.19) | 0.154 | 0.97 (0.93, 1.02) |
| ***P* interaction** | | 0.44 |  |  |  |  |  |
| Model was adjusted for age, sex, alcohol drinking status, smoking status, physical activity, education level, BMI, eGFR, TC, SBP, FBG, lipid-lowering and antihypertensive drug use. The models for participants with diabetes were in addition adjusted for glucose-lowering drugs. The *P*-values were generated using the Cox proportional hazards regression models. | | | | | | | |
| Abbreviations: BMI, body mass index; SBP, systolic blood pressure; DBP, diastolic blood pressure; eGFR, estimated glomerular filtration rate; FBG, fasting blood glucose; TC, total serum cholesterol; TTR, time in target range. | | | | | | | |

| **Table S7. HR (95% CI) of stroke according to SBP-TTR in individuals with or without diabetes** **with the adjustment for proteinuria. (n=**28,591**)** | | | | | | | |
| --- | --- | --- | --- | --- | --- | --- | --- |
|  | | **TTR Group** | | | | ***P* for trend** | **Per 10-point** |
|  |  | **>0% to 25%** | **>25% to 50%** | **>50% to 75%** | **>75% to 100%** |  |  |
| **Stroke** | |  |  |  |  |  |  |
| Diabetes | HR (95% CI) | 1.00 (reference) | 1.06 (0.86, 1.31) | 0.93 (0.73, 1.19) | 0.65 (0.50, 0.85) | 0.005 | 0.96 (0.94, 0.99) |
| No diabetes | HR (95% CI) | 1.00 (reference) | 0.88 (0.76, 1.01) | 0.75 (0.64, 0.88) | 0.62 (0.53, 0.73) | <0.001 | 0.95 (0.93, 0.96) |
| ***P* interaction** | | 0.02 |  |  |  |  |  |
| **Ischemic stroke** | |  |  |  |  |  |  |
| Diabetes | HR (95% CI) | 1.00 (reference) | 1.01 (0.81, 1.26) | 0.84 (0.65, 1.10) | 0.67 (0.51, 0.88) | 0.004 | 0.96 (0.93, 0.99) |
| No diabetes | HR (95% CI) | 1.00 (reference) | 0.85 (0.73, 0.99) | 0.76 (0.64, 0.90) | 0.61 (0.51, 0.72) | <0.001 | 0.95 (0.93, 0.96) |
| ***P* interaction** | | 0.03 |  |  |  |  |  |
| **Hemorrhagic stroke** | |  |  |  |  |  |  |
| Diabetes | HR (95% CI) | 1.00 (reference) | 1.29 (0.69, 2.39) | 1.73 (0.93, 3.23) | 0.37 (0.13, 1.11) | 0.563 | 0.98 (0.90, 1.06) |
| No diabetes | HR (95% CI) | 1.00 (reference) | 1.24 (0.85, 1.79) | 0.78 (0.49, 1.25) | 0.77 (0.49, 1.21) | 0.175 | 0.97 (0.93, 1.02) |
| ***P* interaction** | | 0.70 |  |  |  |  |  |
| Model was adjusted for age, sex, alcohol drinking status, smoking status, physical activity, education level, BMI, eGFR, TC, SBP, FBG, proteinuria, lipid-lowering and antihypertensive drug use. The models for participants with diabetes were in addition adjusted for glucose-lowering drugs. The *P*-values were generated using the Cox proportional hazards regression models. | | | | | | | |
| Abbreviations: BMI, body mass index; SBP, systolic blood pressure; DBP, diastolic blood pressure; eGFR, estimated glomerular filtration rate; FBG, fasting blood glucose; TC, total serum cholesterol; TTR, time in target range. | | | | | | | |

| **Table S8. Sub-Hazard Ratios (95% CI) of stroke according to SBP-TTR in individuals with** hypertension **using the competing risk model. (n=**28,591**)** | | | | | | | |
| --- | --- | --- | --- | --- | --- | --- | --- |
|  | | **TTR Group** | | | | ***P* for trend** | **Per 10-point** |
|  |  | **>0% to 25%** | **>25% to 50%** | **>50% to 75%** | **>75% to 100%** |  |  |
| **Stroke** |  |  |  |  |  |  |  |
| Diabetes | SHR (95% CI) | 1.00 (reference) | 1.05 (0.85, 1.30) | 0.92 (0.72, 1.17) | 0.65 (0.50, 0.85) | 0.003 | 0.96 (0.94, 0.99) |
| No diabetes | SHR (95% CI) | 1.00 (reference) | 0.86 (0.75, 1.00) | 0.73 (0.62, 0.85) | 0.59 (0.50, 0.70) | 0.934 | 0.94 (0.93, 0.96) |
| **Ischemic stroke** | |  |  |  |  |  |  |
| Diabetes | SHR (95% CI) | 1.00 (reference) | 1.01 (0.80, 1.26) | 0.84 (0.65, 1.08) | 0.67 (0.51, 0.88) | 0.004 | 0.96 (0.93, 0.99) |
| No diabetes | SHR (95% CI) | 1.00 (reference) | 0.84 (0.72, 0.97) | 0.74 (0.63, 0.88) | 0.59 (0.49, 0.70) | <0.001 | 0.94 (0.93, 0.96) |
| **Hemorrhagic stroke** | |  |  |  |  |  |  |
| Diabetes | SHR (95% CI) | 1.00 (reference) | 1.23 (0.67, 2.29) | 1.63 (0.90, 2.95) | 0.34 (0.11, 1.05) | 0.354 | 0.97 (0.90, 1.04) |
| No diabetes | SHR (95% CI) | 1.00 (reference) | 1.20 (0.82, 1.75) | 0.74 (0.46, 1.19) | 0.72 (0.45, 1.13) | 0.091 | 0.97 (0.92, 1.01) |
| Model was adjusted for age, sex, alcohol drinking status, smoking status, physical activity, education level, BMI, eGFR, TC, SBP, FBG, lipid-lowering and antihypertensive drug use. The models for participants with diabetes were in addition adjusted for glucose-lowering drugs. | | | | | | | |
| Abbreviations: BMI, body mass index; SBP, systolic blood pressure; DBP, diastolic blood pressure; eGFR, estimated glomerular filtration rate; FBG, fasting blood glucose; TC, total serum cholesterol; TTR, time in target range. | | | | | | | |


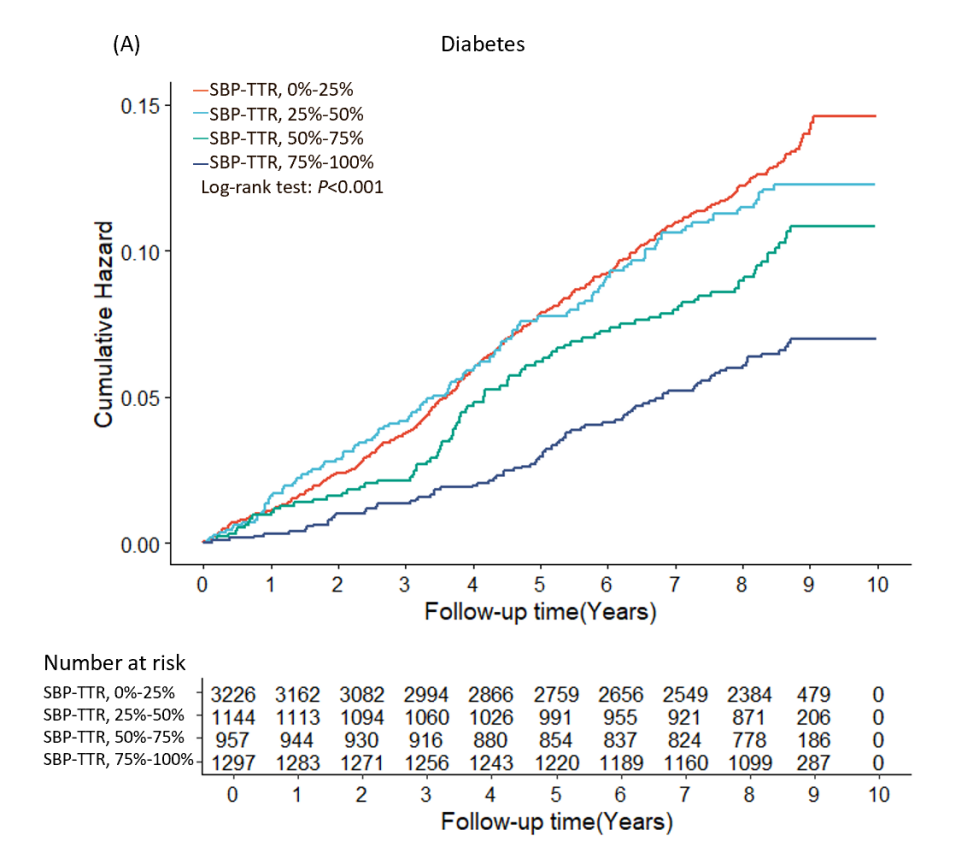

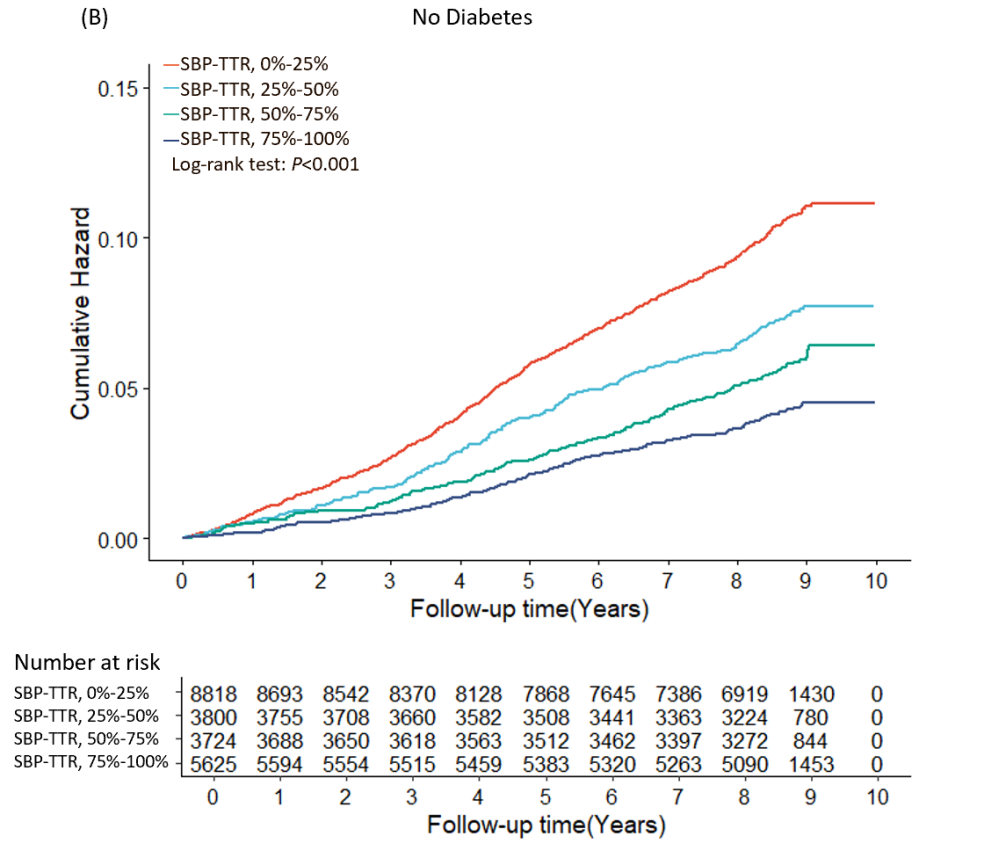


**Figure S1.** Kaplan-Meier curves of cumulative incidence of stroke in individuals with or without diabetes. The figure shows the cumulative risk of stroke in individuals with diabetes (A), without diabetes (B) in groups categorized by TTR. Group 1: SBP-TTR >0% to 25%; Group 2: SBP-TTR >25% to 50%; Group 3: SBP-TTR >50% to 75%; Group 4: SBP-TTR >70% to 100%.
